# Remarks on That Form of Moral Insanity Called Dispsomania, and the Legality of Its Treatment by Isolation

**Published:** 1858-07

**Authors:** David Skae

**Affiliations:** Physician to the Royal Edinburgh Asylum for the Insane


*Remarks on that Form of Moral Insanity called Dispsomania, and the Legality of its Treatment by Isolation.* By DAVID SKAE, M. D., F. R. C. S., Physician to the Royal Edinburgh Asylum for the Insane.* (Read at the Medico-Chirurgical Society, January 20, 1858.)

A man is a drunkard who gets drunk at the festive board—who seeks occasions for getting conveniently drunk without interfering with his ordinary occupations—who takes a few days' drinking at a time when he has plenty of money, and returns to his duties and employment when he has finished his superfluous cash and his "ramble," or "spree," as it is called among the humbler classes in Scotland. Some men, on the other hand get systematically and regularly drunk every day after dinner, some every night before going to bed, and will perform their daily duties with propriety and efficiency. There is a large class who drink continuously, and consume large quantities of stimulants and seem to have a constant craving for them, which they satisfy by constant potations; yet they do not come under the denomination of insane drinkers; they exercise a certain amount of self-control, a sufficient amount of restraint upon their appetites not to allow the quantity which they drink to affect their aptitude for business or driving a good bargain. These are the decorous drunkards of society, and some of them are perhaps the hardest drinkers of any.

The effects of intoxicating drinks vary very much, as is well known, in different individuals according to the temperament or some peculiarity in the constitution. Some men get hilarious and others stupid; some become amorous and others maudlin in their cups; and some, again, become pugnacious, and even dangerous. It has been often observed, that men who have suffered from severe blows or injuries of the head become very violent, and even maniacal, when intoxicated. Such persons as these frequently come under the notice of the legal authorities, for acts of violence committed while intoxicated. But, in these cases, if the individual has voluntarily become intoxicated, knowing that in that state he is liable to be dangerous, he is justly held amenable to punishment for acts of violence committed when drunk. The responsibility of a person of this kind for any of the consequences of his getting drunk, knowing what may ensue, has been compared to that of a person firing a gun out of a window into a crowded street, who would unquestionably be pun-

[* The propriety of any attempt to treat obstinate drunkenness by forcible confinement, is a question of great and increasing importance to the profession and to the public. We have, therefore, made large extracts from the able article pubished by Dr. Skae, in a late number of the Edinburgh Medical Journal.—*Ed.*]

ished for the effects which resulted from his folly. It would be a tedious task to describe all the different effects and kinds of drunkenness. It may suffice to say, that in all cases of mere drunkenness, the characteristic feature, as distinguishing them from insane drinking, is the preservation of the power of self-control. In the mere drunkard, there is a method in his madness; it is a systematised or regulated, or at least a voluntary habit. A morbid craving may be, and is generated, after drunkenness becomes a habit; but it is regulated, as to the time, and mode, and extent, and frequency of gratifying it, by certain considerations—such as a regard to outward decency, to the duties of life, or to the calls of business. And it continues to be so regulated, perhaps by a very low standard, and by one which is gradually made lower and lower; but still the habit is regulated and controlled as long as the person is merely a drunkard, and until the habit degenerates into a disease.

The principal, the diagnostic feature of this disease— that of insane drinking, dipsomania, or oinomania—is on the other hand, the absence or loss of the power of self-control or self-regulation. Persons affected with this form of moral insanity do not drink from the pleasure which the social board affords, but, on the contrary, will not unfrequently preserve a certain amount of decorum while in company. Neither do they drink for the pleasure which the wine gives them; they will drink any kind of intoxicating stuff. Nor do they drink at a convenient time and place, and only occasionally; they drink as often as they can, and whenever they can, and as much as they can; their craving for stimulants is incessant and incontrollable; no considerations of self-respect, no regard to public opinion, or common decency, or domestic ties, or religion or the certainty of impending ruin, degradation, or even the fear of death, can prevent their drinking till they can drink no longer . Such persons will often deplore their fatality, their inability to control ther desires, and will say, with tears in their eyes, as some have done, that if hell were yawning at one side of them, and a bottle of brandy standing at the other, they would drink. although the next moment they were to be thrown into the bottomless abyss. In fine, such persons drink because they cannot help it; and if they really cannot help it, they must be regarded as no longer responsible agents—as therefore insane, and proper objects for being restrained and protected against themselves.

The loss of self-control is, indeed, the most essential condition of almost all cases of insanity. The most constant symptoms of insanity are those referable to altered affections, perverted desires, and morbid propensities. Delusions, so much dwelt upon by some writers, particularly among legal authorities, as essential to insanity, are but its accidental concomitants like hallucinations of the senses. Every form of madness, from acute mania down to dementia, may be met with where there are no delusions. In mania there may be a morbid exaltation of all the emotions and passions over which self-control is entirely lost, and the individual riots in unrestrained laughter, singing, anger, destructiveness, and violence. The loss of control may extend to the thoughts and ideas, which succeed each other with extraordinary rapidity and give rise to incessant talking and incoherence. In partial insanity, one particular emotion or desire may be morbidly excited, and we may find a deep melancholy and depression without any delusion—a hopeless but abstract gloom, leading, by the total overthrow of the power of self-control, to an act of suicide. There may be, as has been fully established by numerous well authenticated cases, a morbid impulse to destroy life, without any delusion, an impulse over which the affected person loses all control, and under which he commits some homicidal act. The morbid excitment of the sexual appetite only becomes insanity when the person loses self-control, and the victim of satariasis or nymphomania outrages all considerations not alone of morality, but of ordinary decency and the common instincts of humanity,

It is, in fact, the loss of control over morbidly excited passions, whether of an exalted or depressing kind, that constitutes insanity, and distinguishes it from ordinary excitement or depression. So it is with insane drinking; it is the loss of control over the morbid appetite that distinguishes it from ordinary drunkenness, and constitutes it a form of moral insanity.

I prefer the term moral insanity, in describing this disease, to the terms oinomania or dipsomania, for three reasons: First, because neither of these names conveys a correct idea of the disease, which is not an insane thirst, nor a thirst for wine only, but may consist in a morbid craving for stimulants or narcotics of any kind; second, because it is under the denomination of moral insanity that this disease is recognized by the commissioners in lunacy for England; and thirdly, for the most important reason of any, namely, that the craving for stimulants is generally only one of many symptoms of moral perversion which characterize the disease, and which it is of great importance to recognize, as they render the duties of the medical attendant comparatively easy, when they are met with in any particular case.

Of these other symptoms of moral perversion, the most common is the habit of lying. In almost all cases of this variety of moral insanity, there is a total disregard for truth. Such persons are singularly mendacious. They will resort to every possible device to procure stimulants, to excuse their conduct, to deceive their friends and medical attendant, and will display an ingenuity and fertility in deceit which is truly marvelous. They will become faint, or be in agony with toothache, or tic, or cramp in the stomach, or colic, or dysmenorrhœa, or they will take diarrhœa or hæmorrhage, and be on the verge of dissolutions, unless brandy, wine, or opium is administered. They will pawn every available article of dress, or furniture, or jewelry. They will borrow from servants or cabmen. They will get whisky smuggled home with their clothes from the tailor or the laundress. They will evade the most vigilant surveillance, and tell the most deliberate falsehoods in their attempts to deceive, solemnly appealing to God for their truth. When shut off from the ordinary sources of stimulation, they will sometimes resort to almost any thing in order to relieve their craving. I have known a young and delicate lady, being prevented getting wine or spirits, and deprived of red lavender, lavender water, and eau de cologne, take creosote, vinegar, vitriol, and tobacco.

Allied to this disregard of truth is the total denial, often insisted upon by such persons, of their habit of over indulgence. They will very frequently disavow most solemnly having ever exceeded the bounds of strict temperance in their use of stimulants; and, if admitting at all that they ever have been the worse of drink, they will blame some other person or circumstance as the cause of it. The wife will blame the husband; the husband, the accident of meeting on one particular occasion some one, or being engaged in some circumstances which rendered it unavoidable. And in this way, one or two undeniable overt acts of intemperance are conceded, when it cannot be avoided, while the habit or the loss of self-control is generally indignantly denied, except at moments of remorse.

Accompanying this disregard of truth, there are often other indications of moral perversion in the insane drunkard, such as extreme licentiousness, or a propensity to theft, or a delight in fomenting quarrels and creating mischief by leading other parties into trouble.

Again, this disease is very frequently met with in persons presenting certain mental peculiarities which are natural to them, that is, congenital. It is by no means uncommon to find it develop itself in youth, who have always been regarded as having a want about them, to use a vernacular phrase. They have never made progress in their education like others of their age—have never, perhaps, been able to learn to spell, and so forth; and, although illiterate and ignorant when they come to manhood, are often very vain, and at the same time credulous and silly. Or, again, they have been characterized, even from childhood, by certain perversities of disposition, such as a delight in cruelty to animals, disregard of personal appearance, love of solitary and gratuitious destructiveness, extreme obstinacy and selfishness, or a violent and mutinous temper upon slight occasion.

Lastly, the disease is very frequently hereditary; and it will be found, on enquiry, that a grand father, or mother, or one or more brothers, have died of delirium tremens, or have otherwise shown the fatal propensity. The constancy with which the hereditary predisposition to this disease is transmitted, is most remarkable, and, in a vast majority of cases can be traced either through the maternal or paternal side of the family.

To illustrate the truth of these remarks, I have analyzed the record of 86 of the cases of this form of moral insanity which I have had under my care, of which 20 were females and 66 males. I find, as the result of these comparisons, that of the 20 females one-half presented natural peculiarities in their mental constitution, being either of weak minds, of very violent and uncontrollable passions, or subject to hysterical attacks of great violence, combined in some instances with great moral depravity.

Of the males, 30, or nearly one-half, were naturally of weak mind, or presented some mental peculiarity, such as silly vanity, general depravity of disposition, and in some cases, considerable talent, but combined with eccentricity or some marked peculiarity. In both sexes, the disregard of truth was a common feature in most cases.

In regard to hereditary predisposition, it is difficult generally to trace this in a public hospital, partly because many cases come in without any information regarding them at all, and partly because the friends, when they have friends, are generally very solicitous to deny or conceal the existence of a hereditary taint in the family, and will not unfrequently positively deny that any other member of the family is insane, while, perhaps, at the time there may be another in one asylum and a sister in another. Notwithstanding these difficulties, I find the hereditary predisposition distinctly traced in 32 cases out of the 86.

Of the females, this hereditary taint was found to exist in 10 out of 20. One had a brother insane; one a mother half witted; one had two cousins who died of intemperance; another had a father and mother who were both intemperate; another had a father, and another a father and brother, who were hard drinkers; three had mothers who died from the effects of intemperance; one had two sisters, both prostitutes and drunkards, one of whom committed suicide; and another had a brother, father mother, all intemperate, and the latter of whom committed suicide.

Of the males, two had brothers affected with the same disease in the asylum, and three brothers who had the same disease, but were not sent to any asylum; one had repeatedly attempted suicide, and inherited, with his whole family, a loathing of life, which at times affected all the members of his family. Four had brothers who suffered from other forms of insanity; two had each two brothers insane; three had insane sisters; two drunken fathers; one a drunken father and brother, and one a father and grand mother, drunkards; and of several others it was admitted that insanity was in the family, although the relationship of the members affected was not ascertained.

Before discussing the question of treatment, and isolation in an asylum or special establishment, it is neccessary to complete this sketch by enumerating briefly the varieties of this form of moral insanity, and the causes which induce the disease.

Its varieties group themselves naturally into three division—the acute, the periodic, and the chronic forms of the malady.

Under the head of acute, I would include all those cases of incontrollable drinking which occur to persons of previously temperate or regular habits, but in whom the insane craving has been generated under the influence of some accidental cause—such as the novelty and excitment of a new sphere of duty, and the temptations of new associates and habits, all combining to lead to intemperance; or the depressing effects of some overwhelming calamity, or of some debilitating accident, or disease, or other agency, such as excessive hœmorrhage, protracted nursing, fever, and such like. It is well known that the use of stimulants, begun and indulged, in under any of these circumstances, has gradually merged, in many instances, into an inordinate and uncontrolable craving, and an insatiable and destructive use of them.

Cases of this kind, however, if taken in time, and treated judiciously and firmly, are generally curable without recourse to any step for depriving the patient of his personal freedom.

Periodic or recurrent attacks of the disease are generally dependent upon some constitutional or hereditary peculiarity. Sometimes they occur at the critical age in females, and preserve a periodic form coinciding with the menstrual period. Sometimes they arise from injuries of the head. In many cases the cause is obscure.

Cases of this kind are less amenable to treatment than the former, particularly where there is a hereditary predisposition. They hardly justify confinement, however, or isolation from society. Where this has been tried, it has generally been found that the uncontrollable impulse comes back at its accustomed period, when the imposed restraint has been removed. Nor does this form of the disease interfere materially, in many cases, with the duties of life. Many individuals have distinguished themselves in literature, or in professional or mercantile life, who have been known for a long term of years to retire periodically into the privacy of their own chamber, and, after indulging this morbid appetite to satiety for a week or two in their voluntary seclusion, to reappear again on the stage of life, and pursue their usual avocations with credit and success. Such cases are perhaps rare, and more frequently it happens that the recurrence of the periodic form gradually degenerates into the chronic variety of the disease, the intervals becoming shorter and the attacks longer, until the intervals cease altogether, and a chronic disease or a fatal issue ensues.

It is the chronic form of this disease which is the least curable and most troublesome to manage. Here the craving for stimulants, brought on perhaps by indulgence and irregular habits, operating upon a constitution hereditary predisposed, becomes constant, insatiable and uncontrollable; and the daily or hourly indulgence suffers only now and then a temporary check by illness induced by it, by delirium tremens, or outbursts of mania.

I believe it is agreed by all parties, that this form of the disease can only be treated effectually by prolonged complete abstinence from all stimulants. This has been attempted in two ways; First, by boarding the patient in some house where, in consequence of its remoteness from places where ardent spirits or other stimulants can be procured, or where, by the stringent rules of the house, and the watchfulness of those connected with it, a barrier may be placed against attempts to gratify the morbid craving. Of the first kind is the boarding-house mentined by Dr. Christison in Skye, where for many years patients of this kind have been sent. Of the latter is the house of refuge in Edinburgh, which has been used for a number of years as a reformatory school for patients of the humbler classes affected with this disease. In addition to these houses, a number of individuals—clergyman, medical men and others, in different parts of the country—have been in the habit of taking boarders affected with this malady, with the view of curing them. In none of these places can the principle of treatment be efficiently carried out. The parties having charge of the patients have no legal authority to detain them, or to interfere with their personal freedom; and the consequence is, that they either leave before a sufficient length of time has elapsed to do any good, or they evade the surveillance under which they are placed, and manage to keep up the craving for stimulants by a constant system of smuggling and secret indulgence. It consists with my experience that this has been the result in all the institutions and houses of the kind mentioned, which I know of, in this country; and I believe every one will agree with me, who has had much experience in the treatment of this deplorable malady, that, in its chronic and confirmed form, complete absence from stimulants can only be effected by depriving the patient of his personal freedom.

In the present state of the law, this can only be done by obtaining a warrant for the detention of the patient in an asylum for the insane.—And this method of treatment is now very commonly adopted in well marked and obstinate cases. I think some medical men, particularly in the country and in provincial towns, still entertain doubts as to the propriety of granting certificates of insanity in such cases. But the scruples of such are gradually wearing away, as the disease becomes better known and more fully recognized as a variety of moral insanity. It is now found, accordingly, that in all the asylums of England, Ireland and Scotland, such, cases are constantly admitted, and regarded both by the medical superintendents, commissioners of lunacy, and the sheriffs of our counties, as the proper objects of asylum restraint.

The principal ground, however, upon which it is contended that some new legislative enactment is required for the treatment of the cases of moral insanity under consideration, is that they cannot be detained long enough in asylums, under the present laws, for the purpose of effecting a cure of the disease. In regard to this point, I must confess that the difficulties I have had chiefly to contend with are such as could be obviated by no new act of legislation. Such patients, when removed too soon, have been removed almost invariably by their own relatives, or on the advice of their own medical attendants. When they recover from the immediate effects of uncontrolled drinking, they appear so sane, they make so many plausible promises, and they are so full of self-reliance, that they generally succsed in persuading their friends that they ought to have a trial; or they try the art of intimidation—threaten to quarrel with their friends and cut them off without a shilling in their wills, unless they take them out, or threaten the medical attendants who consigned them to the asylum with actions of damages, unless they are immediately liberated, and so succeed in getting the one party or the other to recommend their liberation, rather than encounter the threatened alternatives. I

cannot call to my recollection a single instance in which any such case of moral insanity has been ordered out of confinement by the legal authorities contrary to my advice, and before I considered the patient was justly entitled to a trial. I am quite willing to admit that, in many of the cases I have had under my care, the sheriff might have intimated his opinion that they ought to have a trial by being set at large, had his interference not been forestalled by that of the friends; but I feel confident that if such cases by premature liberation, had relapsed and been sent back, their detention would not have been interfered with during a very considerable period of probation. And admitting, as I do, the necessity of a pretty long period of confinement, extending even to one or two years, or even more, for the cure of confirmed and chronic cases of this moral insanity, I cannot but admit at the same time the justice and propriety of giving patients of this class a trial, after their first confinement, at the end of three or six months, if they give any thing like a reasonable hope of doing well, and if their powers of self-control have been tested with favorable results. It has been my practice, and I believe it has been that of the physicians of other chartered asylums, to test the powers of self-control of such patients, after they have been sufficiently weaned, by abstinence, from their morbid cravings—to permit them to go beyond the asylum on their parole, to visit their friends, to spend a day or two with their relatives, or even to lodge for a week or two in the neighborhood; and if they display, under those circumstances, the ability to control their appetites and regulate their conduct, then I consider myself bound to liberate them; while, on the other hand, if they break through the restraints imposed upon them by their own voluntary pledges, and are unable to keep their promises and purposes of temperance, I consider myself entitled to prolong their period of confinement, and in doing so I have never yet been interfered with by the arm of the law.

The practice in other parts of the empire may be different—the views which some sheriffs, or the views which our newly appointed commissioners in lunacy may take of such cases, may be different; but I do think, as the real nature of this deplorable disease becomes more fully known, the administration of the existing laws must expand, so as to permit all that can be properly and wisely asked as to the duration of time required for the effectual treatment of such cases, with a view to their complete cure of a morbid, and uncontrollable, and self-destroying appetite.

In fine, if the disease which we call moral insanity, or dipsomania, is not really a form of insanity, it would be very dangerous to legislate on the subject; but if it is a form of insanity—and of this, we have no doubt—the existing laws regarding the insane ought to meet the requirements for its treatment; and, by a liberal and enlightened administration of those laws, I do not doubt they will be found to do so, without the necessity for new legislation. If any thing more is wanted let some enterprising individual, or company, with a moderate capital, license a country-house surrounded with an agreeable landsape, and with grounds affording extended walks or drives, and the amusements of fishing and shooting; let the establishment command all the sources of recreation which a well arranged asylum possesses; and let it be designated by some appropriate and agreeable name—not "asylum," nor "inebriate asylum," like the American institution of the kind, which, I believe did not succeed; let the patients of this class be sent to that sanatorium under a warrant of the sheriff as insane; and let them be treated according to the most approved principles of our art, under the direction of an enlightened physician, with an efficient staff of trustworthy attendants; and let patients be taken at different rates of board, suitable to their means, and I doubt not most of the difficulties at present felt in dealing with this disease, if not all, will be successfully met, and that such an establishment would soon be distinguished for its usefulness, and prove a valuable source of revenue to its proprietors.—*Virginia Medical Journal.*

